# Associations between Dietary Intake and Attention Deficit Hyperactivity Disorder (ADHD) Scores by Repeated Measurements in School-Age Children

**DOI:** 10.3390/nu14142919

**Published:** 2022-07-16

**Authors:** Su-a Ryu, Yean-Jung Choi, Hyojin An, Ho-Jang Kwon, Mina Ha, Yun-Chul Hong, Soo-Jong Hong, Hyo-Jeong Hwang

**Affiliations:** 1 Department of Food and Nutrition, Kyung Hee University, Seoul 02447, Korea; sua9336@naver.com (S.-a.R.); ddottori@naver.com (H.A.); 2 Department of Food and Nutrition, Sahmyook University, Seoul 01795, Korea; yjchoi@syu.ac.kr; 3 Department of Preventive Medicine, Dankook University College of Medicine, Cheonan 31116, Korea; hojang@dankook.ac.kr (H.-J.K.); minaha@dankook.ac.kr (M.H.); 4 Department of Preventive Medicine, Seoul National University College of Medicine, Seoul 08826, Korea; ychong1@snu.ac.kr; 5 Department of Pediatrics, Childhood Asthma Atopy Center, Humidifier Disinfectant Health Center, Asan Medical Center, University of Ulsan College of Medicine, Seoul 05505, Korea; sjhong@amc.seoul.kr

**Keywords:** dietary intake, mental health, children, attention deficit hyperactivity disorder, nutrients

## Abstract

Attention deficit hyperactivity disorder (ADHD) is a common psychiatric disorder in school-age children and adolescents. However, the reported associations between ADHD and single nutrient intake are inconsistent. The aim of the study was to investigate the relationships between dietary intake changes and the prevalence of ADHD over time with repeat measurements using data from the Children Health and Environment Research (CHEER). To assess changes over time, we used data obtained in 2006 and 2008 (Phases _1_ and _2_). In this study, there were 2899 children aged 8 years or older in Phase _1_ and 2120 children aged 9 years or older in Phase _2_ from Korea, and the ADHD scores and dietary intake of 1733 children in Phases _1_ and _2_ were used in the final analysis. The YN group refers to children whose disease had improved in Phase _2_, and the NY group refers to children diagnosed with ADHD in Phase _2_. A notable within-group result was the increase in vegetable protein (*p* = 0.03) in the YN group. A between-group comparison showed that significant changes in nutrient intake could be confirmed most in the NY group, and the YN group tended to have a lower nutrient intake than the NY group. In the correlation of changes in nutrient intake and three subtypes (combined, AD, and HD), the total fat (*p* = 0.048) and animal protein (*p* = 0.099) showed a positive correlation with the prevalence of AD. Vegetable iron (*p* = 0.061 and *p* = 0.044, respectively), zinc (*p* = 0.022 and *p* = 0.007, respectively), vegetable protein (*p* = 0.074), and calcium (*p* = 0.057) had inhibitory effects on ADHD and its subtype. In conclusion, management of dietary and nutritional status should be considered to ameliorate ADHD and its subtypes in school-age children, and these relationships require further exploration in other settings.

## 1. Introduction

Mental health is an important issue regardless of age, but children’s physical and mental states are particularly important, as they affect education and employment [1] and persist into adolescence and adulthood in a sizable majority of afflicted children [2]. Attention deficit hyperactivity disorder (ADHD) is a common childhood psychiatric disorder found in school-age children and adolescents [3], and it has been recognized as a specific disease since the 1970s. It is classified into a type in which the attention deficit type is dominant, a type in which hyperactivity and impulsivity are dominant, and a complex type in which both symptoms are present [4,5]. The reported prevalence of ADHD varies and is dependent on age and the tools used for assessment but is estimated to range between 6.7% and 15.5% [6,7,8,9]. Epidemiological studies and meta-analyses have reported that approximately 5–12% of school-age children and adolescents experience ADHD [10,11,12,13,14,15,16]. Globally, the prevalence of ADHD was found to be 5.6% in a previous systematic review. In another systematic review, the combined outcome was a prevalence of 7.2%. A recent meta-analysis of the total prevalence of ADHD among children in India showed a total prevalence of 63.2 cases per 1000 children in an integrated setting [17]. Moreover, this proportion has increased steadily over the past decades [8]. For children and adolescents in the United States, the prevalence of ADHD diagnosis significantly increased from 6.1% in 1997–1998 to 10.2% in 2015–2016. However, reports of increased prevalence are controversial [18,19,20,21].

The causes of ADHD are multifactorial and involve genetic and environmental factors [8,22,23]. Although ADHD is considered a genetic disorder, attention has been paid to the potential roles of nutrient deficiency and unhealthy diets in its pathogenesis [8,22,24]. Adequate nutrition positively impacts child growth and development, whereas asymmetric nutrition slows growth and brain function, and reduces learning and memory capacities [25]. Scientists have focused on heavy metal exposure, chemical exposure, lifestyle, and psychosocial factors [8,23]. Nutrients such as *ω*-3 [3], zinc, iron, selenium, copper, lead [26], vitamin D [27], iodine [6,28], food consumption [22,29], food security [30], dietary pattern [8,31,32], and dietary behavior (e.g., frequency of overeating, eating pace, skipping breakfast, and eating fast food) [4,16,33,34] have also received considerable attention. Although iodine, iron, zinc, choline, vitamin B, folic acid, and vitamin D are important for brain development, dietary intake and biosynthesis differ among individuals. One method used to treat ADHD involves high-protein allergic food restrictions, such as nuts, eggs, milk, chocolate, and wheat [35], and dose–response relationships have been reported between diet quality and health-related quality of life (HRQoL) in children [36,37,38]. However, the reported associations between ADHD and single nutrient intake are inconsistent.

Diet is a major source of essential nutrient intake, and there is growing evidence linking essential nutrients to ADHD. A recent meta-analysis examining dietary patterns of children and their diagnosis or symptoms of ADHD found that children who scored lower on healthy eating patterns and higher on unhealthy eating patterns had a higher risk of diagnosis or symptoms of ADHD [39]. Although the role of diet in behavioral problems in children is controversial, the relationship between some nutritional factors and ADHD has been consistently found. In a recent study of Spanish preschoolers, the ADHD group was less attached to health patterns than the control group, positively associated with Western patterns, and adhered to a sweet food pattern similar to the control group [40]. Another study of the US pediatric population reported that increased fruit and vegetable intake was inversely related to the degree of attention deficit [41]. These findings suggest that dietary intake is associated with inattentive symptoms and emotional dysregulation in children with ADHD. A recent population-based birth cohort study reported that suggesting an overall more inflammatory diet with a higher maternal dietary inflammatory index during pregnancy may have a protective effect on ADHD behavioral symptoms in boys [42].

Research on child mental health is being conducted in various fields, but study designs have often been cross-sectional or case–control, and few large-scale, repeated investigations have been performed on ADHD. Although the association between nutritional status in children and ADHD has also been analyzed, diet-associated practices change, especially in children, and may be more variable than those of adults; thus, repeated measurements are required when studying children. Therefore, an examination of the relationships between nutrient changes and the prevalence of ADHD over time might provide a realistic perspective of such relationships. We undertook this study to confirm that dietary intake is associated with ADHD in school-age children.

## 2. Materials and Methods

### 2.1. Study Design

The data used in the present study were obtained from the Children Health and Environment Research (CHEER), a prospective nationwide cohort study conducted to examine the association between environmental agents and children’s health in the Korean Environmental Health Survey [43]. This study was approved by the Institutional Review Board of the Dankook University College of Medicine and University of Ulsan College of Medicine (2005-15, 2006-26, 2007-54, 0810-051, 0904-026, and 01094-04-02).

### 2.2. Study Participants

**Figure 1:**
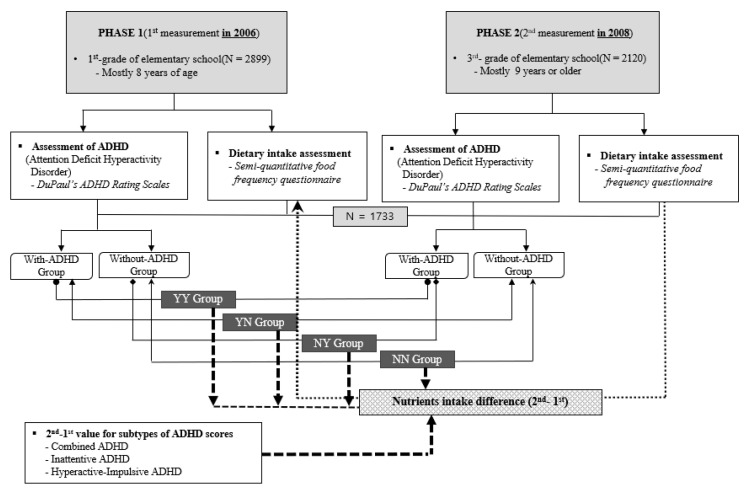
Flow chart of study 2.3. Primary outcome measure: ADHD scores.

CHEER was launched in 2005 to investigate and compare the health status of children in their early childhood (7–12 ages) living under different environmental conditions and those of children exposed to environmental toxins. To assess changes over time, we used data obtained in 2006 and 2008 in a prospective study (termed Phases _1_ and _2_, respectively). Phase _1_ involved 2899 children mostly of grade 1 (approximately 8 years old) at elementary schools, and phase _2_ involved 2120 children of grade 3 (approximately 9 years old and older) at elementary schools. The number of children who took part in the 2006 and 2008 studies was 1733. A total of 1733 children with ADHD scores and dietary intake for Phases _1_ and _2_ were enrolled in the final analysis (Figure 1). NN: Children without ADHD in both Phases _1_ and _2_, YN: Children whose disease improved over time, YY: Children considered to have ADHD in both Phases _1_ and _2_, and NY: Children later diagnosed with ADHD.

At each phase, the Korean version of DuPaul’s rating scale, validated and standardized for Korean children [44], was used to diagnose ADHD. The ADHD rating scale involves summing the scores awarded to responses to 18 questions like the original scale [45,46], where higher scores indicate more severe ADHD symptoms. Questions were awarded 0 to 3 points; thus, the maximum possible score was 0–54 points. Scores > 19 points were considered to indicate the possible presence of ADHD subtypes: attention deficit, hyperactivity, and combined ADHD. In this study, based on this dichotomization, we allocated children to a “with ADHD group” or a “without-ADHD group”. For analysis purposes, we constructed four other groups based on the results obtained in the two tests: the NN, YN, YY, and NY groups. The YN group was composed of children considered to have ADHD in Phase _1_ but not to have ADHD in Phase _2_. On the other hand, the NY group consisted of children who were considered non-ADHD in Phase _1_ but had been diagnosed with ADHD in Phase _2_. The NN group consisted of children without ADHD in both Phases _1_ and _2_, and the YY group consisted of children considered to have ADHD in both Phases _1_ and _2_.

### 2.3. Secondary Outcome Measure: Dietary Intake

Dietary intake was documented using a semi-food frequency questionnaire (SFFQ), and parents were asked to fill it out. The SFFQ used in this study was developed as a useful method to investigate the dietary intake of preschool children in Korea in previous studies [47]. The FFQ reliability (how similar values can be measured when repeated measurements are taken) correlation coefficient was 0.6–0.8, and the validity was 0.3–0.6. To assess dietary intake, we composed a seven-category, 94-food-item SFFQ. The seven categories were cereals (rice, bread, and noodles), meat and fish (including processed meat), legumes, vegetables, fruits, milk and dairy products, and snacks. Consumption of the 94 food items was provided by mothers based on a 1-month recall. Frequency of intake was assessed as the number of times (from one to nine) items were consumed daily, weekly, or monthly, and the size of the portion was assessed as small, medium, or large. CAN PRO 4.0 (Computerized Nutrient Analysis provided by the Korean Nutrition Society) was used to calculate nutrient compositions.

### 2.4. Other Measurements

Data on age, sex, height, and weight were collected, and body mass indices (BMIs) were calculated by dividing weight (kg) by height squared (m^2^). Socioeconomic characteristics, including parental marital status (single, married, or divorced (widowed/separated)), parental education level (high school, college, university, or graduate school), and family income (low income: <KRW 2 million/month, middle income: KRW 2–5 million/month, or high income: >KRW 5 million) were also collected.

### 2.5. Statistical Analysis

Demographic and socioeconomic variables were analyzed using descriptive statistics, and a paired t-test was used to analyze the nutrient intake. In addition, the dietary intake for Phases _1_ and _2_ was analyzed for boys and girls. A t-test was used to analyze the association between nutrient intake and the with/without ADHD group at each phase. Intergroup differences between the NY, YN, YY, and NN groups were assessed using the least square method (LS-mean). Age, sex, family income, marital status, and calorie intake were included as control variables [48,49,50,51]. To ensure no selection bias, ANOVA was used to compare continuous variables according to the central limit theorem [52]. Statistical analyses were performed using SAS version 9.4 (SAS Institute, Cary, NC, USA).

## 3. Results

Table 1 displays the general characteristics of the study participants in Phase _1_ (N = 2741) and Phase _2_ (N = 2102). The mean participant’s age was 7.4 years (SD 0.6) at Phase _1_ and 9.4 years (SD 0.6) at Phase _2_. Regarding each socioeconomic variable, the percentages of middle income (Phase _1_: 56.7%, Phase _2_: 59.4%), living with parents (Phase _1_: 83.9%, Phase _2_: 87.5%), and parents with high school education (father: 47.4%, mother: 60.7%) were the highest (*p* < 0.001). The prevalence of ADHD was significantly higher in Phase _1_ (12.1% vs. 9.1%; *p* < 0.001) and significantly higher in boys than in girls during both phases (Phase _1_: boys 18.4% and girls 7.1%; and Phase _2_: boys 13.4% and girls 5.6%) (both *p* < 0.001). The number of children allocated to the NN, YN, YY, and NY groups was 1422 (83.6%), 125 (7.4%), 82 (4.8%), and 72 (4.2%), respectively (*p* = 0.005). The YN, YY, and NY groups included significantly more boys than girls.

The results of the nutrient analysis are presented in Table 2. The mean calorie of Phase _1_ was approximately 103–105% (boys 1757.4, girls 1584.6 kcal) of the estimated energy requirements (EER); Phase _2_ was 83–89% (boys 1753.6, girls 1603.2 kcal) (energy intake level/EER*100). In the crude model, every significant difference was higher in boys; however, in the energy-adjusted model, every significant difference was found to be higher in girls. In Phases _1_ and _2_, in the energy-adjusted model, the intake levels of vegetable calcium, phosphorus, total iron, iron, sodium, potassium, zinc, vitamin A, β-carotene, vitamin C, folate, and vitamin E, including fiber, were higher in girls than in boys.

**Figure 2:**
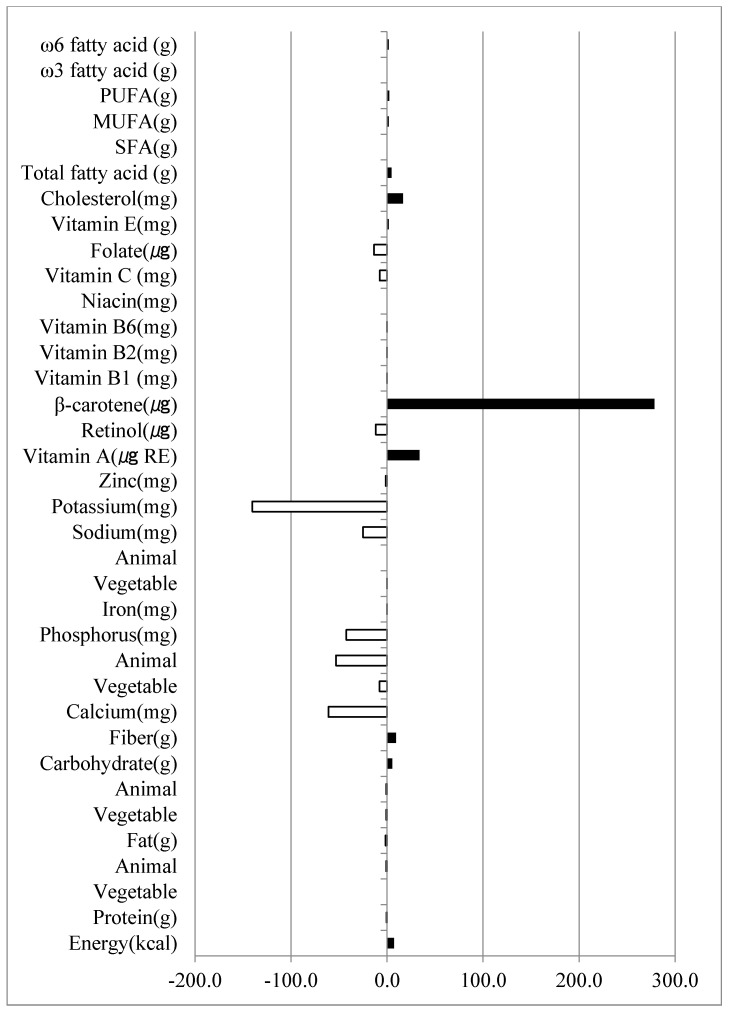
Nutrient intake differences based on the Phase _2_–Phase _1_ intake values. Paired T-test crude model, genders not separated.

Figure 2 shows the differences in nutrient intake between Phases _1_ and _2_ (second–first intake level) (sex not separated). The total calcium, vegetable calcium, animal calcium, phosphorus, potassium, zinc, vitamin B2, retinol, vitamin C, and folate levels in Phase _1_ were significantly higher than those in Phase _2_. In contrast, Phase _2_ showed higher fiber, vitamin A, carotene, vitamin E, cholesterol, total fatty acids, monounsaturated fatty acids (MUFA), polyunsaturated fatty acids (PUFA), ω-3, and ω-6 levels than Phase _1_.

Table 3 shows the differences in nutrient levels between the with- and without-ADHD groups (crude model). Interestingly, a significant difference in the number and type of significant nutrients was observed according to the phase. In Phase _1_, only folate showed a significant difference between the with- and without-ADHD groups (with-ADHD group: 186.0 ± 103.5, without-ADHD group: 203.6 ± 119.4 µg, *p* < 0.02). In Phase _2_, all significant nutrient intake levels were higher in the with-ADHD group. In particular, there was a difference in the energy intake of 300 kcal between the with- and without-ADHD groups (with-ADHD group: 1905.9, without-ADHD group: 1657.5 kcal, *p* = 0.005).

Table 4 shows the differences in nutrient intake (Phases _2–1_) for each of the four groups (NN, YN, NY, YY). In the energy-adjusted model, significant nutrient levels were observed in the NN and YN groups. In the NN group, the levels of total fat (*p* < 0.001), vegetable fat (*p* < 0.001), zinc (*p* = 0.004), and animal protein (*p* < 0.02) in Phase _1_ were higher than those in Phase _2_, and vegetable protein (*p* = 0.002) levels in Phase _2_ were higher than those in Phase _1_. The level of vegetable protein (*p* = 0.03) in Phase _2_ in the YN group was higher than that in Phase _1_ (despite being significant, the difference in vitamin B1 levels in the YY group was small).

**Figure 3:**
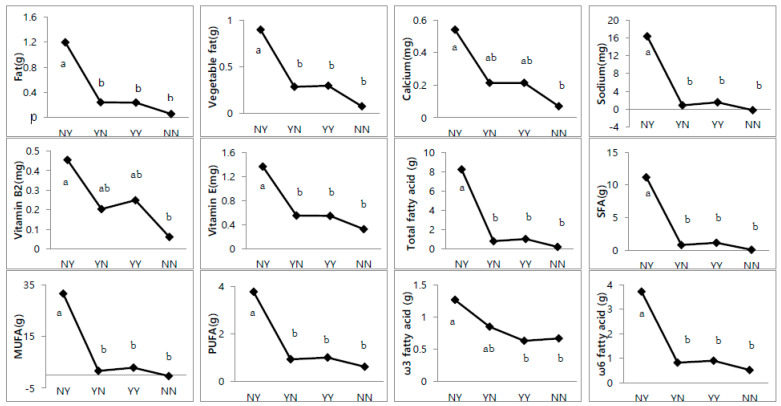
Association between nutrient intakes based on the Phase _2_–Phase _1_ intake values and four ADHD groups. Data followed by the same lowercase letter indicate no significant difference at *p* < 0.05.

As shown in Figure 3, a between-group comparison revealed significant differences in the nutrient intake (Phases _2–1_) of NN, YN, NY, and YY. The most significant change in nutrient intake could be confirmed in the NY group compared to the other groups. The changes in total fat, vegetable fat, calcium, sodium, vitamin B2, vitamin E, total fatty acids, SFA, MUFA, PUFA, ω-3, and ω-6 were higher in the NY group than in the other groups. In contrast, the changes in calcium and vitamin B2 were the smallest in the NN group compared to the other groups.

Table 5 lists the correlation between the nutrient intake (Phase _2_–Phase _1_) and ADHD scores. Negative correlations were observed between the combined ADHD and vegetable iron (*p* = 0.061) and zinc (*p* = 0.022). A negative correlation appeared between the AD and vegetable protein (*p* = 0.074) and iron (*p* = 0.044). In contrast, a positive relationship was observed among the animal protein (*p* = 0.099), total fat (*p* = 0.048), and AD. In the case of HD, a negative correlation was observed between calcium (*p* = 0.057) and zinc (*p* = 0.007).

## 4. Discussion

Many studies have examined the association between ADHD, food, and dietary factors. On the other hand, most studies examined the association in a case–control study, one measurement, or a small-scale study. To the best of the authors’ knowledge, few large-scale studies have used repeated measurements with a prospective design. This study focused mainly on observing the changes in nutrient intake and the occurrence of ADHD through repeated measurements for a certain period of time among children aged 7–12. In this study, the prevalence was significantly different according to measurement time; 12.1% (Phase _1_) and 9.1% (Phase _2_) (*p* < 0.001) mean children aged 9–11 years and 6–8 years, respectively. As already mentioned, the range of ADHD prevalence was rather wide: 3–10% in school-age children [6,53]. Therefore, despite the 3.0% decrease in prevalence in Phase _2_ in this study, it is within the acceptable range. However, it is necessary to analyze the association between the change in dietary intake and prevalence after adjusting for all variables that can have an effect, except for nutrients. The prevalence of ADHD is generally higher in men than in women. A clear 3:1 to 4:1 male predominance over females has been observed in a community-based sample of young individuals [3]. This study showed the same tendency: approximately 3:1 (Phase _1_) and 2.6:1 (Phase _2_) (*p* < 0.001).

In both Phases _1_ and _2_, the BMI of the with-ADHD group was higher than that of the non-ADHD group; however, this difference was not significant. Although ADHD and BMI have been studied in association with obesity [3,7,10], the obesity rates were generally higher in the ADHD group than in the normal group; however, this study did not show similar results.

Family income and parental marital status were correlated with ADHD diagnosis. In general, but not necessarily, the food security of low-income households is lower than that of high-income households. Food insecurity related to adequate food intake affects pediatric ADHD symptoms, with possible effects lasting into adulthood [30]. A recent meta-analysis by Wu et al. reported that household food insecurity was significantly associated with lower HRQoL, physical, and psychosocial functioning among children [36]. In the four groups (NN, YN, NY, and YY), the number of children without ADHD in Phases _1_ and _2_ (i.e., the NN group) was higher in the high-income class than in the other groups (high-income class: 88.8%, middle-income class: 84.3%, and low-income class: 79.2% (*p* = 0.013). In contrast, the number of children who continued to be diagnosed with ADHD was the highest in the low-income class (high-income class: 3.9%, middle-income class: 2.8%, and low-income class: 4.1% (*p* = 0.013)). The results of this study can be interpreted collinearly, as in the study by Wu et al. However, before concluding, there were subtle issues. The YN group showed improved symptoms over time, which was the highest in the middle-income group. The number of children who presented with symptoms in Phase _2_ (i.e., the NY group) was the highest in the low-income class. Therefore, for a detailed analysis, an adjusted model is necessary to include the variable of family income. With respect to children with divorced parents, the number of without-ADHD over the whole study period (NN group) was the lowest (Phase _1_: 5.2%, Phase _2_: 6.3%), and those with a diagnosis of ADHD over the whole study period (YY group) were the highest (Phase _1_: 17.3%, Phase _2_: 19.8%) (*p* = 0.000). However, an adjusted model will be necessary for detailed analysis to include the variable of parental marital status because the number of children with a single parent was the highest in the NN group (Phase _1_: 3.2%, Phase _2_: 2.6%).

The estimated mean energy intake was 1757.4 kcal, 1753.6 kcal (boys in Phases _1_ and _2_), 1584.6 kcal, and 1603.2 kcal (girls in Phases _1_ and _2_). Phases _1_ and _2_ showed higher calorie intake in girls than in boys. The age at classification was different in the KDRI in this study. The age of the children who were included in this study ranged from 8 years to over 9 years, and the children’s age of classification of KDRIs consisted of 6–8, 9–11, and 12–14 ages. In addition, age comparisons were unsuitable. Therefore, this study compared the age group that included the most children (Phase _1_: 8 years, Phase _2_: over 9 years) with that of the KDRIs.

In this study, differences in nutrient intake levels in each group (Phases _2–1_) were compared within and between groups to provide a more detailed correlation than comparisons at single time points. The within-group results showed that the NN and YN groups had significant nutrients. In the NN group, children who had never been diagnosed with ADHD during the study period had a higher level of total fat (*p* < 0.0001) and vegetable fat (*p* < 0.0001) in Phase _1_ than in Phase _2_. The YN group, with-ADHD in grade 1 of elementary school and without-ADHD in grade 3 of elementary school, had a higher level of vegetable protein in Phase _2_ than in Phase _1_. No significant differences were observed between the other groups. A notable result in the within-group comparison was the increase in vegetable protein content in the YN group. In the YN group, children who showed improved symptoms over time were observed to have a higher intake of vegetable protein in the third grade than in the first grade of elementary school. This result supports the alleviative effect of vegetable proteins on ADHD symptoms.

A between-group comparison showed that children who were diagnosed with ADHD in Phase _2_, the NY group, showed significantly more nutrients than the other groups. These included vegetable fat (*p* < 0.001), calcium (*p* = 0.015), sodium (*p* < 0.001), vitamin B2 (*p* = 0.007), vitamin E (*p* < 0.001), total fatty acids (*p* < 0.001), SFA (*p* < 0.001), MUFA (*p* < 0.001), PUFA (*p* < 0.001), ω-3 (*p* = 0.029), ω-6 (*p* < 0.001), and total fat (*p* < 0.001). These results showed a positive number, which means that the level of Phase _2_ was higher than that of Phase _1_. In contrast, the difference in the levels of calcium (*p* = 0.015) and vitamin B2 (*p* = 0.007) in the NN group was the lowest compared with that in the other groups. Comparing only two groups, the NY and YN groups, with a change in disease presence, the YN group tended to have a lower nutrient intake than the NY group, suggesting the possibility of prevention and treatment of ADHD due to dietary intake. In a number of systematic reviews analyzing the effects of different nutritional interventions on behavioral symptoms, the authors found a relationship between ADHD and an unhealthy diet, where children and adolescents with ADHD preferred more palatable and poorly nutritious foods [21,39,54]. They ate more high-sugar and high-fat foods, and consumed less fruits, vegetables, whole grain cereals, and high-quality protein foods. Another author found that children and adolescents with ADHD were less adherent to the Mediterranean diet and had fewer healthy habits in their diet than the control group. These studies did not provide data on age, duration, or ADHD subtype, or the sample size was not large enough. They also did not take into account factors that could influence diet, such as gender, parental education level and occupation, medications, IQ, psychological complications, and autism spectrum disorder comorbidities [17,40,55].

In the correlation result of the difference in nutrient intake (Phases _2–1_) and three-subtype ADHD (combined- ADHD, AD, and HD), positive or negative nutrient intake affected the prevalence of ADHD and its subtype. Total fat (*p* = 0.048) and animal protein (*p* = 0.099) levels were positively correlated with the prevalence of AD. Vegetable iron, zinc, protein, and calcium had inhibitory effects on the prevalence of ADHD and its subtypes. Vegetable iron was negatively correlated with ADHD (*p* = 0.061) and AD (*p* = 0.044). Zinc levels were negatively correlated with ADHD (*p* = 0.022) and HD (*p* = 0.007). According to Chou et al., children with ADHD tend to consume less calcium and vitamin B2 than healthy controls [29]. In addition, a case–control study of 592 Chinese children with ADHD revealed that blood zinc levels were negatively related to ADHD (*p* = 0.003) [32]. Iron is an essential cofactor for several functions, such as oxygen transport, immune function, cellular respiration, neurotransmitter metabolism, and DNA synthesis. Zinc is also an essential trace element necessary for cellular functions involved in the metabolism of neurotransmitters, melatonin and prostaglandins. Epidemiological studies show that iron and zinc deficiencies are common nutritional deficiencies worldwide and play an important role in nerve function, malaise appetite, and mood swings. Altered levels of iron and zinc are associated with worsening and progression of ADHD. In a recent systematic review of randomized clinical trials of the role of iron and zinc in the treatment of ADHD in children and adolescents, it is unclear whether changes in nutritional levels in blood tests mediate treatment outcomes in children with ADHD taking mineral supplements, but lower levels of iron and zinc have been observed in children diagnosed with ADHD compared to healthy controls. Importantly, consistent with our findings, the results of this systematic review support the existence of a subgroup that may particularly benefit from treatments that include dietary zinc and iron supplements [40,56].

Two measurements over 3 years revealed a significant difference in nutrient intake, but it was difficult to observe the probable association with the higher prevalence in Phase _1_ than in Phase _2_ and in boys rather than girls. The nutrients with higher levels in Phase _1_ were total calcium, vegetable calcium, animal calcium, phosphorus, potassium, zinc, vitamin B2, retinol, vitamin C, and folate. In contrast, the levels of fiber, vitamin A, carotene, vitamin E, cholesterol, total fatty acids, MUFA, PUFA, *ω*-3, and *ω*-6 were higher in Phase _2_ than in Phase _1_. In Phases _1_ and _2_, in the energy-adjusted model, the levels of carbohydrates, fiber, vegetable calcium, phosphorus, total iron, iron, sodium, potassium, zinc, vitamin A, retinol, β-carotene, vitamin C, folate, and vitamin E were higher in girls than in boys. Although differences in prevalence according to sex and measurement time were observed, it was difficult to confirm some nutrients’ facilitatory or controlling effects on ADHD prevalence. Interestingly, the number and type of nutrients that were significant in nutrient intake analysis according to the diagnosis of ADHD in each phase were determined. Folate was the only significant nutrient in Phase _1_. The level of the without-ADHD group was higher than that of the with-ADHD group (without-ADHD group: 203.6 ± 119.4, with-ADHD group: 186.0 ± 103.5 µg, *p* = 0.02). On the other hand, in Phase _2_, every significant nutrient level was higher in the with-ADHD group: vegetable protein, animal protein, total fat, animal fat, calcium, iron, zinc, vitamin B1, B2, B6, niacin, cholesterol, fatty acids, SFA, MUFA, PUFA, and energy. That is, if only based on these results, it can be concluded that when participants of this study population were in grade 1 in elementary school, folate had a positive effect on the disease, and when in grade 3, many nutrients, including calories, had a negative effect on the disease. These results were identified because the ADHD risk group had more sweet food intake and more rice than the general group. Abnormally high intake of carbohydrate foods increases insulin secretion, and epinephrine secretion is achieved as a mechanism for reducing the hypersecreted insulin level. Due to the nature of epinephrine, hypersecretion of epinephrine causes hyperactivity and memory loss, which is the same as a study that showed ADHD symptoms [54,55,57]. Globally, studies have shown that ADHD is associated with Western dietary patterns, diets high in added sugar, and refined carbohydrates. Additionally, ADHD and emotional symptoms are inversely proportional to the quality of a better diet, which reflects adherence to Mediterranean and Dutch dietary patterns [41].

In addition, as a result of analyzing the relationship between ADHD and dietary intake, it was found that the ADHD risk group had poorer eating behavior than the normal group. In other words, they tended to eat less frequently, eat a lot at once, and eat quickly [58]. The results were similar to those of the study showing that the ADHD diagnosis group had fewer breakfasts, high ramen consumption, frequent unhealthy eating, and low milk intake compared to the normal group. This trend was also observed in foreign studies. A study in the United States found that the ADHD group was more likely to have difficulty controlling intake than the general group [59], and a study of female college students in Germany also found that female college students with ADHD symptoms had a higher risk of bulimia than the general group [60]. Judging by these findings alone, people with ADHD symptoms, regardless of age, generally had worse eating behaviors than those without.

In a case–control study with children aged 8.1–9.8 in China, patients with ADHD had lower intakes of calcium and vitamin B2 [29]. In addition, although not a nutrient analysis, in Taiwan, in a study composed of 216 cases with ADHD and 216 controls from elementary school children, the ADHD group had lower serum folate, vitamin B6, and ferritin levels, and higher MUFA and SFA concentrations than the control group [22]. They suggested that a nutrient-poor diet leads to suboptimal blood nutritional biochemistry, which, in turn, contributes to the development of ADHD. However, the single causal role of specific nutritional deficiencies in children with ADHD or the role of specific dietary nutrients in the management of this disorder have not yet been elucidated. Managing diet and nutritional status can be considered as an adjuvant therapy to improve ADHD symptoms in children, and further investigation is needed to clarify whether nutritional risk factors may lead to ADHD symptoms among children.

This study has several limitations and strengths. The few similar references to analytical methods regarding the four groups of ADHD impeded an accurate analysis of the comparisons and differences, and the blood nutrient levels related to changes in nutrient intake as the children grow old were not considered. To study the correlation between blood nutrient concentrations and ADHD symptoms, blood nutrient concentrations should be analyzed regularly and monitored in future trials. Additionally, people with certain dietary deficiencies may benefit more from nutritional supplementation, but it should be the focus of future research to test this hypothesis. In this regard, future clinical trials should include information on the concomitant status and use of other nutritional supplements that may have a masking effect to identify patient populations more likely to respond to nutritional supplementation. Family income and living with parents were found to play an important role in prevalence. Therefore, nutrient analysis is needed to consider these two variables, but the crude or energy-adjusted model was used instead. In addition, a comparison of the between-group differences of four groups revealed a significant difference in some nutrients of the NY and NN groups, but it is necessary to pay attention not only to the difference and intake level at each phase (e.g., comparison with KDRIs, ranking in four groups). Moreover, there may be some recall biases and inaccuracies despite dietary assessments of mothers with a 1-month recall. However, to assess general dietary intake in large studies, SFFQ has been proposed as the easiest and least costly dietary assessment method with low recall bias. The SFFQ used in this study was developed and verified in 2002 in consideration of the dietary intake characteristics of pre-school children in Korea [47]. Finally, the method of ADHD diagnosis may be due to the lack of sensitivity compared with a medical diagnosis.

The prospective and large sample size could be a strength of this study, but the strongest point is the repeated measurement for the same sample over a 3-year period. The human dietary life is variable. It is important to identify predisposing factors for early life modification, such as nutritional factors, especially because the treatment options available to infants and young children are limited, and the long-term effectiveness of treatment is relatively low. If nutrients from a dietary change could positively or negatively influence the prevalence, repeated measurements and observations are necessary for a certain follow-up period. Therefore, the repeated measurements in this study are meaningful. In order to determine whether there is an optimal age for nutritional supplementation and to evaluate the optimal treatment period, future studies should focus on the preventive effect of nutritional supplementation.

## 5. Conclusions

The results of a 3-year-study composed of dietary and ADHD assessments with repeated measurements (twice) showed that the with-ADHD group later (at Phase _2_) recorded higher levels of nutrients, such as total fat, than the without-ADHD group. The same tendency of total fat was positively correlated with ADHD score. Negative correlations were observed between vegetable iron and zinc intake and ADHD scores. Children diagnosed with ADHD later (NY group) showed a larger change in quantity than children whose disease had improved over time (YN group). These results may be the basis for further research into diagnosis and interventions in children with ADHD.

## Figures and Tables

**Table 1:** The characteristics of the study population by the diagnosis of ADHD ^(1)^.

Characteristics by Survey Time											
		Phase _1_ (*n* = 2741)		*p*		Phase _2_ (*n* = 2102)		*p*		Final (*n* = 1733)	*p*
		Mean age 7.4 [SD 0.6] Boys 1348 (51.16%)/Girls 1287 (48.84%)				Mean age 9.4 [SD 0.6] Boys 1069 (50.86%)/Girls 1033 (49.14%)				Boys 882 (50.9%)/ Girls 851 (49.1%)	0.555
Family income (10^4^ KRW)	<200	847 (32.8)		<0.001		479 (27.0)		<0.001		-	
	200–500	1466 (56.7)				1055 (59.4)					
	>500	273 (10.2)				243 (13.7)					
Marital status of parent	Single	96 (3.7)		<0.001		65 (3.1)		<0.001		-	
	Married	2272 (83.9)				1807 (87.5)					
	Divorced	216 (8.4)				194 (9.4)					
Father’s education level	High school	-				-				796 (47.4)	<0.001
	College									211 (12.6)	
	University									615 (36.6)	
	Graduate school									58 (3.5)	
Mother’s education level	High school	-				-				1013 (60.7)	<0.001
	College									194 (11.6)	
	University									440 (26.4)	
	Graduate school									22 (1.3)	
Prevalence		12.1%				9.1%				-	
		**Total Sample** **(*n* = 2568)**	**Non-ADHD** **(*n* = 2238)**	**ADHD** **(*n* = 330)**	***p***	**Total Sample (*n* = 2094)**	**Non-ADHD** **(*n* = 1894)**	**ADHD** **(*n* = 200)**	***p***		
Gender (M) (%)	Boys	1317	1075 (81.6)	242 (18.4)	<0.001 ***	1062	920 (86.6)	142 (13.4)	<0.001 ***		
	Girls	1251	1163 (92.9)	88 (7.1)		1032	974 (94.4)	58 (5.6)			
BMI (mean ± SD) (kg/m^2^)			16.8 ± 2.5	17.1 ± 2.6	0.0223		18.1 ± 2.9	18.3 ± 3.3	0.2975		
Family income											
Low income			685 (27.1)	123 (4.9)	0.0453		407 (22.9)	70 (4.0)	<0.0001		
Middle income			1271 (50.4)	173 (6.8)			967 (54.5)	87 (4.9)			
High income			243 (9.6)	29 (1.2)			227 (12.8)	16 (0.9)			
Marital status of parents											
Single			79 (3.1)	11 (0.4)	<0.0001		62 (3.0)	3 (0.2)	<0.0001		
Married (living together)			1964 (77.8)	264 (10.5)			1651 (80.2)	149 (7.2)			
Divorced (widowed or separated)			156 (6.2)	51 (2.0)			149 (7.2)	45 (2.2)			
**Characteristics by four ADHD groups**											
		**NN**			**YN**		**YY**			**NY**	***p***
Frequency		1422 (83.6)			125 (7.4)		82 (4.8)			72 (4.2)	0.005
Age in Phase _1_		7.4 [0.6]			7.3 [0.6]		7.4 [0.5]			7.5 [0.8]	-
Age in Phase _2_		9.4 [0.6]			9.3 [0.6]		9.4 [0.5]			9.5 [0.8]	-
Gender	Boys	662 (38.9)			92 (5.4)		64 (3.8)			49 (2.9)	<0.001 ***
	Girls	760 (44.7)			33 (1.9)		18 (1.1)			23 (1.3)	
Family income in Phase _1_	<200	395 (80.6)			37 (7.6)		32 (6.5)			26 (6.2)	
	200–500	870 (84.7)			80 (7.8)		41 (4.0)			43 (3.7)	
	>500	19 (86.4)			1 (4.6)		2 (9.1)			5 (2.8)	
Family income in Phase _2_	<200	282 (79.2)			25 (7.9)		27 (4.1)			22 (6.2)	0.013 **
	200–500	976 (84.3)			92 (5.4)		47 (2.8)			43 (3.7)	
	>500	158 (88.8)			8 (4.5)		7 (3.9)			5 (2.8)	
Marital status in Phase _1_	Single	45 (3.2)			3 (2.4)		2 (2.5)			1 (1.4)	0.001 **
	Married	1293 (91.6)			110 (88.0)		65 (80.3)			64 (90.1)	
	Divorced	74 (5.2)			12 (9.6)		14 (17.3)			6 (8.5)	
Marital status in Phase _2_	Single	36 (2.6)			4 (3.2)		2 (2.5)			0 (0.0)	0.000 **
	Married	1287 (91.2)			108 (86.4)		63 (77.8)			63 (88.7)	
	Divorced	89 (6.3)			13 (10.4)		16 (19.8)			8 (11.3)	

[SD] (%); ** *p* < 0.05, *** *p* < 0.001; ^(1)^ *p*-value by chi-square test.

**Table 2:** Nutrient intake level according to the measurement time and gender.

**Model 1 (Unadjusted)**						
	Phase _1_ (*n* = 1733)			Phase _2_ (*n* = 1733)		
	Boys (*n* = 882)	Girls (*n* = 851)	*p*	Boys (*n* = 882)	Girls (*n* = 851)	*p*
Energy (kcal)	1757.4 ± 742.1	1584.6 ± 673.9	<0.0001	1753.6 ± 849.1	1603.2 ± 850.9	0.0002
Protein (g)	62.4 ± 28.7	56.6 ± 28.2	<0.0001	61.4 ± 30.6	56.8 ± 34.2	0.003
Vegetable	27.7 ± 12.1	25.5 ± 11.4	0.0001	27.8 ± 13.6	26.4 ± 15.0	0.04
Animal	34.6 ± 19.7	31.0 ± 20.3	0.0002	33.6 ± 19.8	30.3 ± 22.1	0.001
Fat (g)	55.0 ± 29.3	49.9 ± 27.3	0.0002	53.4 ± 31.2	48.3 ± 32.6	0.0009
Vegetable	23.9 ± 14.1	22.1 ± 13.0	0.008	23.1 ± 15.4	21.6 ± 16.4	0.05
Animal	31.1 ± 17.9	27.7 ± 16.4	<0.0001	30.3 ± 18.2	26.7 ± 18.1	<0.0001
Carbohydrate (g)	256.3 ± 107.6	230.4 ± 98.0	<0.0001	259.6 ± 126.3	238.0 ± 120.7	0.0003
Fiber (g)	3.6 ± 2.1	3.5 ± 2.1	0.71	13.1 ± 7.5	12.8 ± 8.2	0.43
Calcium (mg)	728.0 ± 459.7	634.2 ± 367.4	<0.0001	659.4 ± 475.2	581.1 ± 394.0	0.0002
Vegetable	199.6 ± 118.7	192.9 ± 115.9	0.23	190.9 ± 134.1	185.9 ± 134.3	0.44
Animal	528.4 ± 397.4	441.3 ± 297.8	<0.0001	468.4 ± 385.5	395.1 ± 315.0	<0.0001
Phosphorus (mg)	15.8 ± 8.9	14.9 ± 8.8	0.04	15.9 ± 10.3	15.2 ± 11.0	0.18
Iron (mg)	9.6 ± 4.7	8.9 ± 4.4	0.004	9.4 ± 5.0	9.0 ± 5.4	0.09
Vegetable	7.0 ± 3.5	6.5 ± 3.3	0.007	6.8 ± 3.7	6.5 ± 3.9	0.20
Animal	2.5 ± 1.5	2.3 ± 1.6	0.01	2.5 ± 1.5	2.4 ± 1.7	0.02
Sodium (mg)	2874.9 ± 1734.5	2799.9 ± 1815.7	0.37	2844.8 ± 1782.9	2780.0 ± 2067.3	0.48
Potassium (mg)	2338.4 ± 1272.3	2173.6 ± 1166.9	0.005	2187.9 ± 1320.5	2043.9 ± 1284.6	0.02
Zinc (mg)	9.5 ± 5.6	9.1 ± 7.8	0.20	8.1 ± 4.0	7.6 ± 4.1	0.002
Vitamin A (μg RE)	605.9 ± 406.6	584.4 ± 400.0	0.26	643.7 ± 462.8	614.2 ± 451.0	0.17
Retinol (μg)	236.3 ± 165.9	201.7 ± 122.9	<0.0001	224.9 ± 149.5	189.6 ± 128.4	<0.0001
β-carotene (μg)	2015.7 ± 1818.6	2103.1 ± 1886.1	0.32	2323.6 ± 2120.0	2350.9 ± 2080.7	0.78
Vitamin B1 (mg)	1.0 ± 0.5	0.9 ± 0.4	<0.0001	1.0 ± 0.5	0.9 ± 0.5	0.0005
Vitamin B2 (mg)	1.3 ± 0.7	1.2 ± 0.6	<0.0001	1.3 ± 0.7	1.1 ± 0.7	<0.0001
Vitamin B6 (mg)	1.4 ± 0.8	1.3 ± 0.8	0.01	1.4 ± 0.8	1.3 ± 0.9	0.07
Niacin (mg)	12.1 ± 6.6	11.1 ± 6.7	0.001	12.0 ± 6.3	11.3 ± 7.7	0.05
Vitamin C (mg)	81.1 ± 68.9	77.7 ± 63.9	0.27	72.7 ± 66.8	70.9 ± 64.8	0.57
Folate (μg)	206.5 ± 123.6	196.3 ± 113.1	0.07	190.5 ± 118.1	185.5 ± 124.5	0.38
Vitamin E (mg)	11.3 ± 7.4	10.5 ± 7.1	0.02	12.9 ± 7.9	12.4 ± 9.5	0.30
Cholesterol (mg)	295.7 ± 175.4	270.5 ± 168.3	0.002	316.2 ± 196.3	283.2 ± 200.1	0.0006
Total fatty acid (g)	28.3 ± 16.8	25.2 ± 13.3	<0.0001	33.1 ± 18.9	29.8 ± 19.4	0.0004
SFA (g)	13.5 ± 8.5	11.9 ± 6.8	<0.0001	14.1 ± 8.6	12.2 ± 7.7	<0.0001
MUFA (g)	9.5 ± 6.0	8.5 ± 4.5	<0.0001	11.4 ± 6.5	10.3 ± 6.8	0.0005
PUFA (g)	5.2 ± 3.2	4.8 ± 2.8	0.006	7.5 ± 4.6	7.2 ± 5.7	0.23
ω3 fatty acid (g)	0.6 ± 0.4	0.5 ± 0.5	0.07	0.8 ± 0.5	0.8 ± 0.8	0.69
ω6 fatty acid (g)	4.6 ± 2.9	4.2 ± 2.5	0.004	6.3 ± 3.9	6.1 ± 4.7	0.22
**Model 2 (Energy-adjusted model)**						
	Phase _1_ (*n* = 1733)			Phase _2_ (*n* = 1733)		
	Boys (*n* = 882)	Girls (*n* = 851)	*p*	Boys (*n* = 882)	Girls (*n* = 851)	*p*
Energy (kcal)						
Protein (g)	59.7 ± 8.8	59.6 ± 9.5	0.64	59.0 ± 8.5	59.2 ± 8.7	0.58
Vegetable	26.6 ± 4.9	27.0 ± 5.0	0.18	26.8 ± 4.8	27.7 ± 5.0	<0.0001
Animal	32.7 ± 10.4	32.8 ± 11.2	0.92	32.2 ± 10.8	31.7 ± 10.2	0.36
Fat (g)	51.9 ± 12.6	52.7 ± 13.8	0.16	50.9 ± 13.3	50.4 ± 12.7	0.36
Vegetable	22.5 ± 6.9	23.2 ± 7.4	0.02	21.9 ± 7.0	22.2 ± 6.9	0.31
Animal	29.3 ± 9.6	29.4 ± 9.9	0.83	29.9 ± 10.6	29.1 ± 9.8	0.09
Carbohydrate (g)	245.1 ± 31.3	244.1 ± 33.9	0.55	244.5 ± 33.1	246.9 ± 31.6	0.12
Fiber (g)	3.4 ± 1.3	3.7 ± 1.4	<0.0001	3.4 ± 0.9	3.6 ± 1.1	<0.0001
Calcium (mg)	684.4 ± 666.0	681.1 ± 661.7	0.80	686.3 ± 296.4	686.3 ± 278.9	0.99
Vegetable	190.6 ± 70.9	201.9 ± 75.7	0.001	189.3 ± 66.8	204.6 ± 87.2	<0.0001
Animal	494.8 ± 476.6	484.1 ± 465.7	0.41	503.3 ± 314.3	492.1 ± 287.1	0.44
Phosphorus (mg)	15.0 ± 4.9	15.7 ± 5.6	0.01	15.1 ± 5.6	16.0 ± 6.7	0.004
Iron (mg)	9.1 ± 1.7	9.4 ± 1.9	0.0004	9.1 ± 1.5	9.5 ± 1.8	<0.0001
Vegetable	6.6 ± 1.5	6.9 ± 1.7	0.001	6.6 ± 1.5	7.0 ± 1.7	<0.0001
Animal	2.4 ± 0.8	2.5 ± 0.9	0.14	2.4 ± 0.8	2.4 ± 0.8	0.48
Sodium (mg)	2761.4 ± 1147.4	2954.7 ± 1319.0	0.001	2753.2 ± 1020.2	1352.9 ± 1352.9	0.0004
Potassium (mg)	2209.1 ± 578.8	2294.9 ± 663.1	0.004	2213.4 ± 562.0	2294.2 ± 646.8	0.005
Zinc (mg)	9.0 ± 2.5	9.5 ± 5.8	0.009	8.9 ± 0.9	9.0 ± 1.0	0.007
Vitamin A (μg RE)	572.4 ± 249.6	619.4 ± 329.0	0.001	572.7 ± 247.4	611.8 ± 264.0	0.001
Retinol (μg)	221.9 ± 104.3	217.8 ± 98.7	0.40	227.8 ± 108.0	216.3 ± 105.9	0.02
β-carotene (μg)	1918.2 ± 1332.6	2192.3 ± 1520.6	<0.0001	1855.8 ± 1199.7	2121.5 ± 1317.0	<0.0001
Vitamin B1 (mg)	1.0 ± 0.1	1.0 ± 0.2	0.99	1.0 ± 0.2	1.0 ± 0.1	0.43
Vitamin B2 (mg)	1.3 ± 0.3	1.2 ± 0.3	0.72	1.3 ± 0.3	1.2 ± 0.3	0.12
Vitamin B6 (mg)	1.3 ± 0.3	1.4 ± 0.3	0.002	1.4 ± 0.3	1.4 ± 0.3	0.004
Niacin (mg)	11.4 ± 2.6	11.6 ± 2.6	0.22	11.4 ± 2.8	11.7 ± 2.6	0.07
Vitamin C (mg)	75.6 ± 72.3	81.4 ± 49.8	0.01	74.5 ± 42.9	82.4 ± 48.4	0.0004
Folate (μg)	195.4 ± 70.5	207.5 ± 73.9	0.0005	194.4 ± 66.0	209.0 ± 77.8	<0.0001
Vitamin E (mg)	10.6 ± 3.8	11.1 ± 4.2	0.004	10.5 ± 3.5	10.9 ± 3.4	0.01
Cholesterol (mg)	283.9 ± 119.9	287.7 ± 126.6	0.52	291.3 ± 127.9	285.4 ± 117.9	0.31
Total fatty acid (g)	26.8 ± 9.0	27.1 ± 9.3	0.40	27.3 ± 9.8	26.8 ± 8.6	0.25
SFA (g)	12.8 ± 5.4	12.9 ± 5.5	0.82	13.0 ± 5.5	12.6 ± 5.2	0.08
MUFA (g)	9.0 ± 3.1	9.1 ± 3.1	0.35	9.2 ± 3.6	9.0 ± 2.9	0.18
PUFA (g)	4.9 ± 1.7	5.1 ± 1.8	0.02	5.0 ± 1.9	5.1 ± 1.8	0.21
ω3 fatty acid (g)	0.5 ± 0.2	0.6 ± 0.3	0.06	0.5 ± 0.2	0.6 ± 0.2	0.10
ω6 fatty acid (g)	4.4 ± 1.5	4.5 ± 1.6	0.03	4.5 ± 1.7	4.6 ± 1.6	0.22

**Table 3:** Nutrient intake level according to the diagnosis of ADHD ^(1)^.

	**Phase _1_**			**Phase _2_**		
	Normal (*n* = 1497)	ADHD (*n* = 207)	*p*	Normal (*n* = 1571)	ADHD (*n* = 158)	*p*
	Mean ± SD	Mean ± SD		Mean ± SD	Mean ± SD	
Energy (kcal)	1674.7 ± 717.9	1652.5 ± 631.6	0.64	1657.5 ± 822.7	1905.9 ± 1087.3	0.005
Protein (g)	59.8 ± 28.7	57.8 ± 24.5	0.27	58.4 ± 31.8	66.3 ± 38.0	0.01
Vegetable	26.8 ± 11.8	25.8 ± 11.3	0.28	26.9 ± 14.0	29.6 ± 17.1	0.05
Animal	33.0 ± 20.2	31.9 ± 16.2	0.37	31.5 ± 20.8	36.7 ± 23.1	0.007
Fat (g)	52.5 ± 28.5	52.8 ± 25.7	0.85	50.1 ± 31.1	59.5 ± 38.8	0.003
Vegetable	23.0 ± 13.6	23.1 ± 13.2	0.91	21.9 ± 15.3	26.1 ± 20.2	0.01
Animal	29.4 ± 17.3	29.7 ± 15.7	0.81	28.1 ± 17.8	33.3 ± 21.1	0.003
Carbohydrate (g)	243.9 ± 104.3	239.3 ± 92.2	0.50	246.0 ± 119.4	279.6 ± 160.3	0.01
Fiber (g)	3.6 ± 2.1	3.3 ± 2.1	0.09	12.8 ± 7.8	13.6 ± 9.0	0.31
Calcium (mg)	688.5 ± 416.8	646.1 ± 405.2	0.16	613.0 ± 417.0	705.5 ± 612.6	0.06
Vegetable	197.6 ± 117.0	187.3 ± 115.8	0.23	187.5 ± 130.1	199.3 ± 169.5	0.39
Animal	490.9 ± 351.7	458.8 ± 347.1	0.21	425.5 ± 339.2	506.1 ± 477.7	0.03
Phosphorus (mg)	15.4 ± 8.8	14.7 ± 8.0	0.24	15.4 ± 10.5	16.5 ± 12.0	0.28
Iron (mg)	9.3 ± 4.6	8.9 ± 4.1	0.15	9.1 ± 5.0	10.0 ± 6.4	0.08
Vegetable	6.8 ± 3.4	6.4 ± 3.2	0.14	6.6 ± 3.7	7.1 ± 4.9	0.22
Animal	2.4 ± 1.5	2.4 ± 1.4	0.55	2.4 ± 1.6	2.8 ± 1.8	0.004
Sodium (mg)	2845.4 ± 1760.7	2738.8 ± 1631.8	0.41	2793.7 ± 1914.2	2985.2 ± 2000.0	0.23
Potassium (mg)	2274.7 ± 1228.6	2135.3 ± 1056.9	0.08	2101.3 ± 1259.7	2287.0 ± 1684.3	0.17
Zinc (mg)	9.3 ± 6.0	9.3 ± 10.9	0.97	7.8 ± 3.9	8.8 ± 5.3	0.02
Vitamin A (μg RE)	599.8 ± 400.1	567.4 ± 405.6	0.27	625.7 ± 448.9	666.7 ± 533.1	0.35
Retinol (μg)	219.9 ± 148.9	220.0 ± 133.5	0.99	203.2 ± 136.1	250.6 ± 173.1	0.001
β-carotene (μg)	2081.4 ± 1837.6	1889.5 ± 1870.1	0.16	2342.1 ± 2071.8	2299.8 ± 2383.9	0.83
Vitamin B1 (mg)	1.0 ± 0.5	1.0 ± 0.4	0.30	1.0 ± 0.5	1.1 ± 0.6	0.005
Vitamin B2 (mg)	1.3 ± 0.7	1.2 ± 0.6	0.23	1.2 ± 0.7	1.4 ± 0.9	0.01
Vitamin B6 (mg)	1.4 ± 0.8	1.3 ± 0.7	0.30	1.4 ± 0.9	1.5 ± 1.1	0.05
Niacin (mg)	11.6 ± 6.8	11.2 ± 5.4	0.34	11.5 ± 6.9	13.3 ± 8.3	0.01
Vitamin C (mg)	80.1 ± 67.4	74.9 ± 57.5	0.23	71.4 ± 63.8	76.2 ± 83.9	0.49
Folate (μg)	203.6 ± 119.4	186.0 ± 103.5	0.02	187.1 ± 118.4	198.2 ± 147.9	0.36
Vitamin E (mg)	10.9 ± 7.2	10.6 ± 6.5	0.45	12.5 ± 8.7	13.9 ± 9.3	0.06
Cholesterol (mg)	284.4 ± 172.9	276.9 ± 152.6	0.51	295.0 ± 197.5	345.2 ± 197.4	0.002
Total fatty acid (g)	26.8 ± 15.3	27.1 ± 14.6	0.84	31.1 ± 19.0	35.7 ± 21.1	0.008
SFA (g)	12.7 ± 7.8	12.9 ± 7.7	0.82	13.0 ± 8.0	15.2 ± 9.7	0.005
MUFA (g)	9.0 ± 5.4	9.2 ± 5.0	0.72	10.7 ± 6.7	12.4 ± 7.1	0.003
PUFA (g)	5.0 ± 3.0	4.9 ± 3.0	0.80	7.3 ± 5.2	8.1 ± 4.9	0.08
ω3 fatty acid (g)	0.6 ± 0.4	0.5 ± 0.4	0.84	0.8 ± 0.7	0.9 ± 0.6	0.27
ω6 fatty acid (g)	4.4 ± 2.7	4.4 ± 2.7	0.88	6.2 ± 4.3	6.8 ± 4.2	0.10

^(1)^*p*-value by T-test.

**Table 4:** Nutrient intake levels for four ADHD groups.

	NN (*n* = 1422)				YN (*n* = 125)			
	Phase _1_	Phase _2_	Phase _2_–Phase _1_	*p*	Phase _1_	Phase _2_	Phase _2_–Phase _1_	*p*
Energy	1664.4 ± 709.9	1650.9 ± 835.	−13.5 ± 888.9	0.567	1585.3 ± 613.9	1735.6 ± 627.6	150.3 ± 702.0	0.018 **
Energy-adjusted								
Protein (g)	59.7 ± 9.0	59.3 ± 8.3	0.4 ± 10.5	0.09	58.6 ± 10.1	57.8 ± 9.2	0.7 ± 11.3	0.43
Vegetable	26.9 ± 4.9	27.3 ± 4.9	−0.4 ± 5.7	0.002 **	26.3 ± 5.5	27.6 ± 5.1	−1.2 ± 6.3	0.03 **
Animal	32.9 ± 10.7	32.1 ± 10.3	0.7 ± 12.8	0.02 **	32.3 ± 11.5	30.2 ± 11.4	2.0 ± 13.2	0.08
Fat (g)	52.4 ± 13.0	50.6 ± 13.0	1.7 ± 16.6	<0.0001 ***	52.8 ± 14.5	49.7 ± 14.2	3.0 ± 18.7	0.06
Vegetable	22.9 ± 7.1	21.9 ± 6.8	0.9 ± 8.8	<0.0001 ***	23.2 ± 8.2	22.5 ± 8.5	0.7 ± 10.9	0.42
Animal	29.4 ± 9.7	29.6 ± 10.3	−0.2 ± 12.2	0.50	29.4 ± 10.1	28.1 ± 11.2	1.3 ± 13.0	0.26
Carbohydrate (g)	244.3 ± 32.1	245.8 ± 32.3	−1.4 ± 40.3	0.16	244.7 ± 35.5	248.1 ± 35.1	−3.4 ± 44.0	0.38
Fiber (g)	3.6 ± 1.3	3.5 ± 1.0	0.07 ± 1.3	0.04	3.4 ± 1.5	3.3 ± 1.0	0.08 ± 1.6	0.57
Calcium (mg)	691.6 ± 281.2	694.2 ± 290.0	−2.5 ± 347.7	0.77	644.8 ± 283.2	623.6 ± 256.1	21.2 ± 325.5	0.46
Vegetable	198.0 ± 73.2	198.7 ± 69.1	−0.6 ± 84.2	0.77	192.6 ± 85.9	186.6 ± 79.5	5.9 ± 100.3	0.50
Animal	496.2 ± 272.6	505.0 ± 308.1	−9.2 ± 354.8	0.32	454.1 ± 279.7	439.5 ± 250.5	14.5 ± 319.2	0.61
Phosphorus (mg)	15.4 ± 5.1	15.7 ± 6.1	−0.2 ± 7.0	0.25	15.5 ± 6.7	14.9 ± 5.8	0.6 ± 7.5	0.34
Iron (mg)	9.3 ± 1.8	9.3 ± 1.6	−0.009 ± 2.0	0.85	9.0 ± 1.9	9.0 ± 1.7	−0.001 ± 2.1	0.99
Vegetable	6.8 ± 1.6	6.8 ± 1.5	−0.02 ± 1.8	0.59	6.6 ± 1.7	6.6 ± 1.6	−0.05 ± 2.0	0.74
Animal	2.4 ± 0.8	2.4 ± 0.7	0.005 ± 1.0	0.85	2.4 ± 0.9	2.3 ± 0.9	0.06 ± 1.0	0.50
Sodium (mg)	2871.0 ± 1214.8	2869.1 ± 1087.7	1.8 ± 1395.2	0.96	2966.5 ± 1583.1	2751.2 ± 1132.6	215.3 ± 1664.5	0.15
Potassium (mg)	2276.8 ± 626.3	2276.6 ± 572.1	0.2 ± 716.3	0.99	2187.5 ± 636.8	2130.9 ± 603.2	56.6 ± 769.8	0.41
Zinc (mg)	9.2 ± 3.3	9.0 ± 0.9	0.2 ± 3.3	0.004 **	8.8 ± 2.5	8.6 ± 0.9	0.1 ± 2.5	0.50
Vitamin A (μg RE)	604.2 ± 298.6	598.1 ± 253.3	6.3 ± 343.4	0.48	568.9 ± 287.5	559.7 ± 241.6	9.1 ± 316.9	0.74
Retinol (μg)	220.0 ± 98.9	221.5 ± 106.5	−1.4 ± 120.0	0.65	216.3 ± 101.7	217.5 ± 111.9	−1.2 ± 126.7	0.91
β-carotene (μg)	2100.0 ± 1461.4	2021.4 ± 1225.1	79.4 ± 1620.6	0.06	1925.5 ± 1459.6	1844.6 ± 1265.6	80.9 ± 1614.5	0.57
Vitamin B1 (mg)	1.0 ± 0.1	1.0 ± 0.1	−0.002 ± 0.2	0.75	1.0 ± 0.1	1.0 ± 0.1	0.002 ± 0.2	0.90
Vitamin B2 (mg)	1.3 ± 0.3	1.3 ± 0.3	0.002 ± 0.4	0.86	1.2 ± 0.4	1.2 ± 0.3	0.03 ± 0.4	0.43
Vitamin B6 (mg)	1.4 ± 0.3	1.4 ± 0.3	−0.008 ± 0.4	0.46	1.4 ± 0.3	1.3 ± 0.4	0.02 ± 0.5	0.59
Niacin (mg)	11.5 ± 2.5	11.6 ± 2.6	−0.06 ± 3.2	0.45	11.4 ± 2.7	11.3 ± 2.5	0.07 ± 3.2	0.79
Vitamin C (mg)	79.7 ± 51.7	79.8 ± 44.4	−0.04 ± 58.8	0.97	74.1 ± 46.8	71.2 ± 46.5	2.8 ± 62.2	0.60
Folate (μg)	204.2 ± 72.8	204.2 ± 68.5	0.01 ± 81.2	0.99	191.7 ± 74.4	189.4 ± 66.6	2.2 ± 88.0	0.77
Vitamin E (mg)	10.9 ± 3.9	10.8 ± 3.4	0.1 ± 4.5	0.26	10.6 ± 4.2	10.3 ± 4.0	0.2 ± 5.3	0.58
Cholesterol (mg)	286.7 ± 124.7	286.8 ± 116.5	−0.03 ± 140.1	0.99	280.8 ± 121.1	285.4 ± 141.5	−4.6 ± 156.8	0.74
Total fatty acid (g)	27.1 ± 9.1	27.3 ± 9.4	−0.1 ± 11.8	0.55	26.4 ± 9.4	25.6 ± 9.1	0.8 ± 11.9	0.41
SFA (g)	12.9 ± 5.4	12.9 ± 5.5	0.01 ± 6.8	0.92	12.4 ± 5.2	12.0 ± 5.3	0.4 ± 6.7	0.49
MUFA (g)	9.1 ± 3.0	9.2 ± 3.4	−0.1 ± 4.2	0.30	9.0 ± 3.4	8.6 ± 3.0	0.3 ± 4.1	0.29
PUFA (g)	5.0 ± 1.7	5.1 ± 1.8	−0.08 ± 2.2	0.15	4.9 ± 1.9	4.8 ± 2.0	0.1 ± 2.4	0.59
ω3 fatty acid (g)	0.6 ± 0.2	0.6 ± 0.2	−0.01 ± 0.3	0.18	0.5 ± 0.3	0.5 ± 0.2	0.009 ± 0.3	0.78
ω6 fatty acid (g)	4.5 ± 1.5	4.5 ± 1.7	−0.08 ± 2.0	0.11	4.4 ± 1.8	4.3 ± 1.8	0.1 ± 2.2	0.51
	**NY (*n* = 72)**				**YY (*n* = 82)**			
	**Phase _1_**	**Phase _2_**	**Phase _2_–Phase _1_**	***p***	**Phase _1_**	**Phase _2_**	**Phase _2_–Phase _1_**	***p***
Energy	1851.1 ± 841.7	1917.2 ± 1301.1	66.1 ± 1409.9	0.692	1755.0 ± 648.3	1919.3 ± 874.6	164.3 ± 893.8	0.100
Energy-adjusted								
Protein (g)	59.1 ± 9.6	58.4 ± 9.6	0.6 ± 10.1	0.57	58.3 ± 8.1	58.8 ± 8.1	−0.4 ± 9.9	0.65
Vegetable	26.7 ± 5.5	26.4 ± 4.4	0.2 ± 6.5	0.76	25.9 ± 5.0	26.2 ± 4.3	−0.3 ± 6.0	0.62
Animal	32.7 ± 10.5	31.9 ± 11.7	0.7 ± 12.5	0.62	32.5 ± 9.2	32.5 ± 9.6	−0.04 ± 12.7	0.97
Fat (g)	51.1 ± 13.5	50.4 ± 12.1	0.7 ± 16.2	0.71	54.2 ± 11.7	52.7 ± 10.6	1.4 ± 14.0	0.36
Vegetable	22.4 ± 6.9	22.1 ± 6.7	0.3 ± 9.6	0.77	23.3 ± 6.7	22.9 ± 7.0	0.4 ± 7.5	0.59
Animal	29.0 ± 9.0	29.1 ± 9.3	−0.1 ± 10.9	0.89	30.7 ± 8.7	30.7 ± 8.5	−0.004 ± 11.6	0.99
Carbohydrate (g)	245.9 ± 34.8	244.8 ± 32.2	1.1 ± 41.5	0.82	241.0 ± 27.0	240.3 ± 27.3	0.7 ± 34.9	0.85
Fiber (g)	3.3 ± 1.1	3.2 ± 0.8	0.1 ± 1.3	0.40	3.4 ± 1.3	3.3 ± 0.8	0.09 ± 1.2	0.49
Calcium (mg)	672.4 ± 302.3	664.4 ± 267.6	7.9 ± 350.2	0.84	665.1 ± 269.3	664.0 ± 269.5	1.1 ± 334.9	0.97
Vegetable	182.4 ± 70.2	171.9 ± 57.0	10.4 ± 81.9	0.28	188.6 ± 57.3	181.1 ± 50.4	7.4 ± 59.9	0.26
Animal	498.1 ± 290.8	498.1 ± 280.3	0.08 ± 369.3	0.99	477.3 ± 269.4	483.6 ± 270.7	−6.3 ± 335.1	0.86
Phosphorus (mg)	14.6 ± 5.1	13.7 ± 3.4	0.9 ± 5.7	0.16	14.3 ± 4.0	15.0 ± 4.5	−0.6 ± 5.7	0.33
Iron (mg)	8.9 ± 1.7	8.9 ± 1.3	0.06 ± 1.9	0.77	8.9 ± 1.7	8.8 ± 1.3	0.1 ± 1.8	0.59
Vegetable	6.5 ± 1.5	6.3 ± 1.3	0.2 ± 1.7	0.32	6.4 ± 1.3	6.3 ± 1.3	0.09 ± 1.4	0.56
Animal	2.4 ± 0.8	2.5 ± 1.0	−0.1 ± 1.0	0.38	2.5 ± 1.3	2.4 ± 0.8	0.03 ± 1.3	0.83
Sodium (mg)	2700.7 ± 1182.0	2539.3 ± 843.4	161.4 ± 1362.0	0.31	2662.0 ± 959.6	2722.1 ± 797.0	−60.0 ± 1099.4	0.62
Potassium (mg)	2102.2 ± 466.5	2059.0 ± 454.8	43.1 ± 507.6	0.47	2145.4 ± 517.7	2145.0 ± 464.9	0.4 ± 659.1	0.99
Zinc (mg)	8.9 ± 2.3	8.8 ± 1.0	0.1 ± 2.2	0.52	10.2 ± 14.4	8.7 ± 1.0	1.5 ± 14.4	0.34
Vitamin A (μg RE)	531.3 ± 214.4	511.6 ± 161.7	17.4 ± 226.0	0.51	575.5 ± 244.3	575.8 ± 309.3	−0.2 ± 309.7	0.99
Retinol (μg)	225.5 ± 139.0	227.3 ± 91.8	−2.8 ± 126.0	0.85	230.6 ± 96.4	242.5 ± 125.9	−11.9 ± 153.9	0.48
β-carotene (μg)	1664.1 ± 969.1	1512.4 ± 707.8	143.9 ± 1011.3	0.23	1897.8 ± 1252.5	1811.9 ± 1584.7	85.9 ± 1437.5	0.58
Vitamin B1 (mg)	1.0 ± 0.2	1.0 ± 0.1	0.03 ± 0.2	0.21	1.0 ± 0.1	1.0 ± 0.2	−0.06 ± 0.31	0.05 **
Vitamin B2 (mg)	1.2 ± 0.4	1.2 ± 0.2	0.02 ± 0.4	0.63	1.2 ± 0.3	1.3 ± 0.3	−0.07 ± 0.4	0.14
Vitamin B6 (mg)	1.3 ± 0.3	1.3 ± 0.2	0.03 ± 0.3	0.37	1.4 ± 0.3	1.4 ± 0.3	−0.01 ± 0.4	0.76
Niacin (mg)	11.2 ± 3.2	11.1 ± 2.3	0.1 ± 2.9	0.61	11.2 ± 2.5	11.8 ± 3.4	−0.5 ± 4.1	0.26
Vitamin C (mg)	67.1 ± 32.0	61.8 ± 29.9	5.1 ± 36.6	0.24	77.2 ± 45.7	72.5 ± 32.8	4.6 ± 53.7	0.43
Folate (μg)	184.4 ± 60.8	183.2 ± 58.1	1.1 ± 64.5	0.87	188.4 ± 62.8	184.4 ± 53.5	3.9 ± 70.4	0.60
Vitamin E (mg)	10.2 ± 4.1	10.1 ± 3.3	0.1 ± 4.9	0.85	11.0 ± 4.8	10.4 ± 3.0	0.5 ± 5.7	0.37
Cholesterol (mg)	294.5 ± 126.6	317.0 ± 157.4	−24.3 ± 145.4	0.16	280.4 ± 99.0	292.6 ± 123.2	−12.1 ± 122.2	0.37
Total fatty acid (g)	26.5 ± 9.8	26.2 ± 8.7	0.2 ± 11.9	0.84	28.5 ± 8.2	27.3 ± 7.2	0.8 ± 11.0	0.48
SFA (g)	12.7 ± 5.8	12.4 ± 4.4	0.2 ± 6.6	0.70	13.7 ± 5.2	13.0 ± 4.3	0.6 ± 6.4	0.35
MUFA (g)	9.0 ± 3.4	8.9 ± 3.0	0.07 ± 4.2	0.87	9.4 ± 2.6	9.3 ± 2.5	0.1 ± 3.7	0.7
PUFA (g)	4.7 ± 1.8	4.8 ± 1.8	−0.07 ± 2.4	0.78	5.0 ± 1.7	4.9 ± 1.5	0.07 ± 2.2	0.75
ω3 fatty acid (g)	0.5 ± 0.3	0.5 ± 0.2	0.02 ± 0.3	0.64	0.6 ± 0.2	0.5 ± 0.2	0.03 ± 0.3	0.37
ω6 fatty acid (g)	4.2 ± 1.6	4.2 ± 1.6	−0.05 ± 2.1	0.82	4.5 ± 1.6	4.4 ± 1.4	0.1 ± 2.0	0.63

** *p* < 0.05, *** *p* < 0.001.

**Table 5:** The correlation of ADHD scores and the differences of nutrients intake measurement (Phase _2_–Phase _1_) ^(1)^.

	Combined ^(2)^				Inattention ^(2)^				Hyperactivity ^(2)^			
	β		SE	*p*-Value	β		SE	*p*-Value	β		SE	*p*-Value
Protein (g) ^(3)^	3.39	±	11.16	0.761	6.51	±	6.71	0.332	−0.86	±	5.66	0.879
Vegetable	−26.29	±	21.03	0.212	−22.64	±	12.64	0.074 *	−5.22	±	10.66	0.625
Animal	7.97	±	9.59	0.406	9.51	±	5.76	0.099 *	0.45	±	4.86	0.927
Fat (g)	11.67	±	8.62	0.176	10.25	±	5.17	0.048 **	2.66	±	4.37	0.543
Vegetable	18.65	±	14.37	0.194	12.77	±	8.63	0.139	5.21	±	7.29	0.474
Animal	8.48	±	11.27	0.452	9.67	±	6.77	0.154	1.34	±	5.71	0.814
Carbohydrate (g)	−3.62	±	3.28	0.270	−3.37	±	1.97	0.088 *	−0.84	±	1.66	0.614
Fiber (g)	−4.28	±	29.33	0.884	0.78	±	17.62	0.965	−7.23	±	14.87	0.627
Calcium (mg)	−0.65	±	0.43	0.131	−0.21	±	0.26	0.412	−0.41	±	0.22	0.057 *
Vegetable	−1.69	±	1.54	0.273	−0.69	±	0.93	0.458	−0.97	±	0.78	0.216
Animal	−0.52	±	0.43	0.224	−0.16	±	0.26	0.535	−0.34	±	0.22	0.116
Phosphorus (mg)	−0.58	±	0.60	0.337	−0.05	±	0.36	0.888	−0.46	±	0.30	0.131
Iron (mg)	−97.53	±	61.84	0.115	−42.69	±	37.17	0.251	−47.39	±	31.37	0.131
Vegetable	−125.07	±	66.58	0.061 *	−80.61	±	40.03	0.044 **	−45.29	±	33.79	0.180
Animal	37.46	±	118.08	0.751	97.38	±	70.95	0.170	−30.35	±	59.84	0.612
Sodium (mg)	0.00	±	0.10	0.979	0.03	±	0.06	0.608	−0.02	±	0.05	0.637
Potassium (mg)	−0.12	±	0.20	0.547	0.00	±	0.12	0.970	−0.11	±	0.10	0.263
Zinc (mg)	−56.55	±	24.65	0.022 **	−23.13	±	14.83	0.119	−33.79	±	12.49	0.007 **
Vitamin A (μg RE)	−0.47	±	0.40	0.239	−0.17	±	0.24	0.490	−0.28	±	0.20	0.169
Retinol (μg)	0.39	±	1.10	0.725	0.61	±	0.66	0.358	−0.05	±	0.56	0.926
β-carotene (μg)	−0.10	±	0.07	0.177	−0.05	±	0.04	0.300	−0.05	±	0.04	0.155
Vitamin B1 (mg)	191.22	±	520.76	0.714	226.78	±	312.73	0.469	−16.35	±	264.10	0.951
Vitamin B2 (mg)	−116.69	±	300.09	0.697	72.34	±	180.25	0.688	−166.18	±	152.13	0.275
Vitamin B6 (mg)	92.33	±	293.33	0.753	160.48	±	176.19	0.363	−23.65	±	148.76	0.874
Niacin (mg)	31.81	±	33.01	0.335	21.59	±	19.84	0.277	13.35	±	16.74	0.425
Vitamin C (mg)	−0.21	±	2.35	0.929	0.60	±	1.41	0.669	−0.72	±	1.19	0.548
Folate (μg)	−1.51	±	1.70	0.373	−0.83	±	1.02	0.415	−0.59	±	0.86	0.494
Vitamin E (mg)	1.93	±	25.05	0.939	8.46	±	15.05	0.574	−2.75	±	12.70	0.829
Cholesterol (mg)	0.56	±	0.94	0.551	0.78	±	0.56	0.166	0.03	±	0.48	0.954
Total fatty acid (g)	6.10	±	11.17	0.585	4.76	±	6.71	0.478	3.85	±	5.66	0.496
SFA (g)	−4.93	±	20.67	0.812	0.87	±	12.42	0.944	−2.65	±	10.47	0.800
MUFA (g)	30.79	±	30.60	0.315	18.25	±	18.38	0.321	19.81	±	15.50	0.202
PUFA (g)	54.31	±	44.50	0.223	32.81	±	26.74	0.220	31.46	±	22.55	0.163
ω3 fatty acid (g)	−5.77	±	261.83	0.982	32.17	±	157.35	0.838	14.40	±	132.74	0.914
ω6 fatty acid (g)	53.42	±	52.47	0.309	30.17	±	31.53	0.339	34.10	±	26.59	0.200

* *p* < 0.1, ** *p* < 0.05, β*1000, SE*1000 ^(1)^ Adjusted for area, child’s gender, father’s education, and second–first total energy intake and the primary variables including child’s age and each combined, inattention, hyperactivity scores, parental marriage status, household income ^(2)^ second–first values for each combined, inattention, hyperactivity scores ^(3)^ second–first values for nutrients.

## Data Availability

The data used to support this study are included within the article.
